# Tri‐domain proteins 27 alleviates ischemia‐reperfusion injury‐induced acute kidney injury by promoting Gli‐like transcription factor 1 expression via the inhibition of polycomb repressive complex 2 activity

**DOI:** 10.1002/ccs3.70046

**Published:** 2025-09-18

**Authors:** Chongxiang Xiong, Haishan Chen, Baoting Su, Li Zhang, Jingxiang Hu, Qiaowen Wang, Shougang Zhuang

**Affiliations:** ^1^ Department of Nephrology The First Dongguan Affiliated Hospital of Guangdong Medical University Dongguan Guangdong Province China; ^2^ Department of Medicine Rhode Island Hospital and Brown University School of Medicine Providence Rhode Island USA

**Keywords:** acute kidney injury, GLIS1, renal ischemia and reperfusion injury, renal tubular epithelial cell dedifferentiation, TRIM27

## Abstract

Dedifferentiated renal tubular epithelial cell (RTEC) proliferation contributes to renal repair following acute kidney injury (AKI) induced by renal ischemia‐reperfusion (I/R) injury (RIRI). However, the fundamental mechanism underlying RTEC dedifferentiation remains unclear. An animal model of RIRI‐induced AKI was established using I/R, and H_2_O_2_‐treated murine (m) RTECs were used as the cell injury model. Pathological changes were assessed by hematoxylin and eosin and periodic acid–Schiff stain staining. Cell viability and migration were assessed using the cell counting kit‐8 and wound healing assays, respectively. Apoptosis was examined using flow cytometry. Molecular interactions were investigated using coimmunoprecipitation and chromatin IP assays. Tri‐domain proteins 27 (TRIM27) expression was reduced in RIRI mice and H_2_O_2_‐treated mouse renal tubular epithelial cells (mRTECs). TRIM27 overexpression enhanced mRTECs dedifferentiation, proliferation, and migration while inhibiting apoptosis. Mechanistically, TRIM27 reduced polycomb repressive complex 2 (PRC2) activity in mRTECs through the mediation of Enhancer of zeste homolog 2 ubiquitination. Further, PRC2 reduced Gli‐like transcription factor 1 (GLIS1) expression in mRTECs by regulating Histone H3 trimethylated at lysine 27 and DNA methylation. TRIM27 overexpression ameliorated RIRI‐induced AKI in mice by enhancing mRTEC dedifferentiation. TRIM27 upregulation alleviates RIRI‐induced AKI by reducing GLIS1 DNA methylation and promoting GLIS1 expression by inhibiting PRC2 activity.

## Graphical abstract

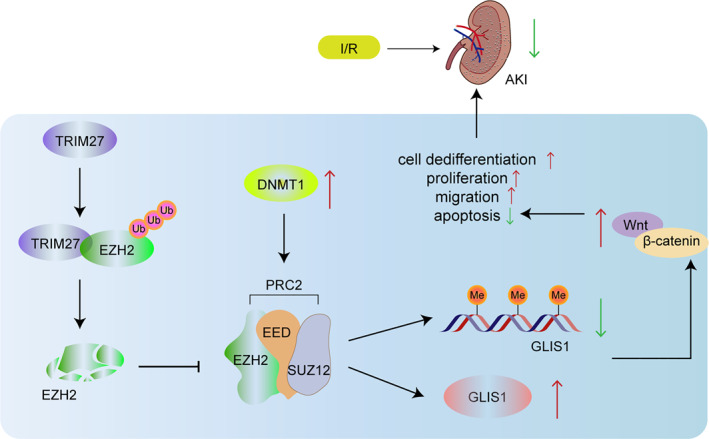

## 1 INTRODUCTION

Renal ischemia and reperfusion injury (RIRI) is a serious disease caused by a series of etiologies, including sepsis, infarction, kidney transplantation, and unilateral nephrectomy. It is characterized by insufficient blood supply, followed by rapid blood flow resuscitation and reoxygenation.^1^ RIRI, associated with high incidence and mortality rates, is one of the most common causes of acute kidney injury (AKI) worldwide.^2^ Unfortunately, despite recent advances in understanding of the pathophysiology of RIRI‐induced AKI, most therapeutic methods commonly fail.^3^ Therefore, efforts to understand the pathophysiological mechanisms are likely to lead to the development of novel medicines for the prevention and treatment of RIRI‐induced AKI.

Renal tubular epithelial cells (RTECs) are extremely sensitive to ischemia/reperfusion injury (IRI), while proximal tubular epithelial cells experience severe injury after IRI.^4^ The remaining RTECs undergo dedifferentiation, proliferation, and migration immediately after RIRI, representing the initial and critical steps in renal regeneration following injury.^5^ Therefore, understanding the process by which damaged tubular cells dedifferentiate would aid in developing a viable therapeutic strategy for RIRI‐induced AKI. Methylation, phosphorylation, and ubiquitination are the main epigenetic modifications that occur during AKI progression.^6^ Polycomb repressive complex 2 (PRC2) is a chromatin‐related methyltransferase comprising three core subunits (Enhancer of zeste homolog 2 (EZH2), Embryonic Ectoderm Development (EED), and Suppressor of Zeste 12 (SUZ12)).^7^ PRC2 dysregulation has been observed in various diseases, and PRC2 is considered a viable target for therapeutic interventions.^8^ Liu et al. revealed that inhibition of the PRC2 subunit EZH2 alleviates renal pyroptosis during RIRI progression.^9^ In addition, EZH2 is highly expressed in murine kidneys with AKI, and inhibiting EZH2 has been shown to reduce tubular injury.^10^ Notably, our previous study showed that PRC2 inhibition protected against cisplatin‐induced AKI.^11^ However, the role of PRC2 in RTEC dedifferentiation during RIRI‐induced AKI remains unclear.

PRC2 upregulation promotes cholangiocarcinoma progression by increasing the methylation levels of the SFRP1 promoter.^12^ Additionally, PRC2 upregulation promotes cell proliferation by increasing DNA methylation of the RASSF1A promoter and reducing its expression.^13^ PRC2 further catalyzes the methylation of histone H3 at lysine 27 (H3K27) via EZH2, with EED and SUZ12 acting as epigenetic “readers,” which is also the mechanism by which PRC2 achieves its complete catalytic activity.^8^ Gli‐like transcription factor 1 (GLIS1) is a subfamily of Krüppel‐like zinc finger transcription factors that regulate multiple cellular processes.^14^ As previously described, GLIS1 alleviates cell senescence and renal fibrosis.^15^ In the current study, CpG islands were predicted to be enriched in the GLIS1 promoter based on the MethPrimer database. In addition, it was predicted that GLIS1 had an Histone H3 trimethylated at lysine 27 (H3K27me3) tag using the Citrome DB website. Nevertheless, the interaction between PRC2 and GLIS1 in the regulation of RTEC dedifferentiation during RIRI‐induced AKI development remains unclear and warrants further investigation.

Tri‐domain protein 27 (TRIM27) is a member of the TRIM protein family which has a zinc‐finger structure, an N‐terminal RING‐finger domain, and a variable domain at the C terminus.^16^ TRIM27 regulates the inflammatory response, apoptosis, cell cycle, cell differentiation, and tumor cell migration.^17^ The role of TRIM27 in the control of kidney injury was also investigated. For example, TRIM27 upregulation promoted glomerular endothelial cell injury in lupus nephritis.^18^ Additionally, TRIM27 upregulation reduced inflammation and apoptosis in HK‐2 cells, and alleviated AKI in mice.^19^ TRIM27 directs key targets for ubiquitination by combining the target with the N‐terminal RING‐finger domain (the signature domain of E3 ubiquitin ligases).^20^ Using the UbiBrowser database, the results of the present study indicates that EZH2 is a TRIM27 ubiquitination substrate. Therefore, we speculated that TRIM27 mediates the ubiquitination and degradation of EZH2 during the progression of RIRI‐induced AKI.

Collectively, we speculated that TRIM27 reduces GLIS1 DNA methylation and increases its expression, subsequently activating the Wnt/β‐catenin pathway by inhibiting PRC2 activity by mediating the ubiquitination degradation of EZH2, thereby promoting the dedifferentiation, proliferation, and migration of RTECs, and ultimately alleviating the development of RIRI‐induced AKI. These findings provide a theoretical foundation for the establishment of new treatment options for RIRI‐induced AKI.

## 2 MATERIALS AND METHODS

### 2.1 Animal experiments

Twenty‐four male C57BL/6J mice (6–8‐week old, 20–22 g) were purchased from SPF Biotechnology Co., Ltd.. After two weeks of adaptation, the mice were randomly divided into sham, RIRI, RIRI + Oe‐NC, and RIRI + Oe‐TRIM27 groups, with six animals in each group. An animal model of RIRI was established, as previously described.^21^ In brief, mice were anesthetized with sodium pentobarbital (60 mg/mL) before undergoing midline laparotomy. Forceps were used to occlude both sides of the renal artery for 30 min. The clamp was withdrawn to allow blood reperfusion. The sham group underwent laparotomy without ligation. Oe‐NC or Oe‐TRIM27 were constructed using the pAdno‐Murine Cytomegalovirus (MCMV)‐EGFP‐3FLAG vector with the MCMV promoter. Adenovirus packaged with Oe‐NC or Oe‐TRIM27 (1 × 10^11^ pfu) were injected into the mice through the tail vein 12 h before RIRI. At 24 h after reperfusion, the mice were euthanized, and kidney tissues and blood were collected. Animal studies were approved by The First Dongguan Affiliated Hospital of Guangdong Medical University.

### 2.2 Hematoxylin–eosin staining

Following fixation, the kidney tissue blocks were embedded in paraffin and cut into 5 μm thick sections. Sections were dehydrated in different concentrations of alcohol and stained with hematoxylin–eosin (HE) (Sigma‐Aldrich). Images were captured using a microscope (Olympus). Histological changes and the number of infiltrating neutrophils were measured in a double‐blind manner in five different regions randomly selected from each sample.

### 2.3 Periodic acid–Schiff staining

Paraffin sections of kidney tissue (5 μm) were prepared and stained with periodic acid–Schiff (PAS) solution (Sigma‐Aldrich). The sections were observed and photographed using an Olympus microscope. A grading system (0–4 points) was used to determine the level of glomerular injury (<25%, 25%–50%, 50%–75%, and >75%).

### 2.4 Measurement of serum creatinine and blood urea nitrogen

Serum creatinine (Scr) and blood urea nitrogen (BUN) levels were measured using kits purchased from StressMarq Biosciences (#SKT‐217, #SKT‐213). All procedures were performed in strict accordance with the manufacturer's instructions.

### 2.5 Terminal deoxynucleotidyl transferase‐mediated dUTP nick‐end labeling staining

Kidney tissues were fixed in 4% paraformaldehyde for 60 min and permeabilized with 0.1% Triton X‐100 for 10 min. The tissues were washed with Phosphate‐Buffered Saline (PBS) and stained with terminal deoxynucleotidyl transferase‐mediated dUTP nick‐end labeling solution (Sigma‐Aldrich). Counterstaining in Diaminobenzidine was subsequently performed.

### 2.6 Cell culture and treatment

Primary mouse renal tubular epithelial cells (mRTECs) were isolated and cultured, as previously reported.^22^ The kidney tissues were dissected and chopped into small pieces, and then digested with 0.75 mg/mL collagenase (Sigma‐Aldrich) for 40 min at 37°C. Tubular tissues were isolated using 31% Percoll gradient and washed with PBS. Cells were grown in Dulbecco's Modified Eagle's Medium (Gibco, MD, USA) supplemented with 10% Fetal Bovine Serum (FBS) (Gibco), 5 pg/mL PGE1, and 10 ng/mL epidermal growth factor with 5% CO_2_ at 37°C. To establish a cell injury model in vitro, mRTECs were incubated with 500 μM H_2_O_2_ (Sigma‐Aldrich) for 24 h, as previously described.^23^ Before H_2_O_2_ treatment, cells were transfected with the short hairpin RNAs (shRNAs) and/or plasmids using Lipofectamine 3000 (Invitrogen) according to the manufacturer's instructions. The shRNAs and plasmids included shRNA of EZH2 or TRIM27 (sh‐EZH2 or sh‐TRIM27), overexpression plasmid of TRIM27 or EZH2 (oe‐TRIM27 or EZH2), and their negative controls, which were purchased from GenePharma.

### 2.7 Cell counting kit‐8 assay

Cells were seeded in 24‐well plates at a density of 2 × 10^4^ cells/well and cultured for 24 h. Cells were then incubated with cell counting kit‐8 solution (10 μL, Yeason, Shanghai, China) at 37°C for 3 h. Absorbance was measured at 450 nm using a microplate reader (Bio‐Rad).

### 2.8 Wound healing assay

Murine (m)RTECs were seeded in the 6‐well plates at a density of 5 × 10^5^ cells/well and cultured for 12 h to allow attachment. Prior to creating wounds, cells were serum‐starved in basal medium (0% FBS) for 10–20 h to minimize proliferation effects on migration. After removing the culture medium, artificial wounds were created using a pipette tip after removing the culture medium. Images were captured at 0 and 24 h.

### 2.9 Cell apoptosis assay

mRTECs were resuspended in the binding buffer and incubated with 10 μL Annexin V‐FITC and 5 μL PI stain (Beyotime) for 10 min. The samples were analyzed by flow cytometry.

### 2.10 Methylation‐specific polymerase chain reaction (PCR)

Genomic DNAs was isolated using the DNA extraction kit (Tiangen, Beijing, China) and subsequently bisulfite‐modified. Primers for methylation and non‐methylation of the GLIS1 promoter were synthesized. For PCR, a methylation‐specific PCR (MSP) reaction system (Takara) was used, which included a template, primer, a PCR buffer, dNTP, and DNA polymerase. MSP products were analyzed by gel electrophoresis.

### 2.11 Coimmunoprecipitation

Cells were lysed and the cell lysates were incubated with Sepharose CL‐4B beads (Sigma‐Aldrich) conjugated with IgG (Abcam, 1:50, ab172730) or TRIM27 (Abcam, 1:50, ab78393) antibody for 4 h. The bound proteins were eluted and examined using western blotting.

### 2.12 Analysis of EZH2 ubiquitination

After lysed, cells were sonicated and incubated in a dilution buffer for 30 min. The supernatant was collected by centrifugation and incubated with an IgG (Abcam, 1:100, ab172730) or an EZH2 antibody (Abcam, 1:30, ab307646) followed by incubation with protein A/G IP magnetic beads for 12 h at 4°C. EZH2 ubiquitination was determined by western blotting using an anti‐Ub antibody (Abcam, 1:1000, ab140601).

### 2.13 Chromatin immunoprecipitation assay

Cells were fixed in 1% formaldehyde for 5 min and lysed. Cell lysates were ultrasonically treated to produce chromatin fragments and incubated overnight with anti‐DNMT1 (Abcam, 1:100, ab19905) or anti‐IgG (Abcam, 1:100, ab172730) antibodies. DNA was immunoprecipitated using protein A/G beads (Thermo Fisher Scientific), and cross‐linking was eliminated. The precipitated DNA was analyzed using qPCR.

### 2.14 Quantitative real‐time PCR

Total RNA was extracted using TRIzol reagent (Invitrogen). cDNA synthesis was performed using a reverse transcription kit (Thermo Fisher Scientific). Quantitative real‐time PCR was performed using the SYBR reagent (Thermo Fisher Scientific). The data were analyzed using 2^−ΔΔCT^ method, with GAPDH as the internal reference gene. The primers used in the study were as follows (5′–3′):

TRIM27 (F): GAAGAAGAGACGGCGGGCAC,

TRIM27 (R): AAGTCGAGCTCCTCAAGGCG;

GLIS1 (F): CCCCTGTCTGTGAGAAGCTG,

GLIS1 (R): GAAAGTCCAGGTCTGAGGGC;

GAPDH (F): AGCCCAAGATGCCCTTCAGT,

GAPDH (R): CCGTGTTCCTACCCCCAATG.

### 2.15 Western blot analysis

Total protein was extracted from cells using RadioImmunoPrecipitation Assay buffer (Beyotime), and the protein concentration was determined using a BCA kit (Thermo Fisher Scientific). Following electrophoresis and membrane transfer, Polyvinylidene Difluoride membranes (Millipore) were blocked in 5% nonfat milk and then incubated with antibodies against Pax‐2 (ab79389), Vimentin (ab92547), TRIM27 (ab78393), EED (ab240650), EZH2 (ab307646), SUZ12 (ab175187), H3K27me3 (ab6002), Histone H3 (ab1791), GLIS1 (PA5‐68892), β‐catenin (ab32572), hexokinase II (HK‐2) (ab209847), pyruvate kinase 2 (PKM2) (PA5‐29339), lactate dehydrogenase A (LDHA) (ab112996), glucose transporter 1 (GLUT1) (ab115730), NADH dehydrogenase (ubiquinone) Fe‐S protein 1‌‌ (NDUFS1) (ab169540), cytochrome c oxidase subunit IV isoform 1 (COX4I1) (ab197658), ATP synthase F1 subunit alpha (ATP5A1) (ab14748), kidney injury molecule‐1 (KIM‐1) (ab233720), β‐actin (ab8226), and GAPDH (ab8245). The membranes were subsequently incubated with a secondary antibody (ab7090) for 60 min. Finally, protein bands were visualized using Enhanced ChemiLuminescence (Beyotime) substrate, and the gray scale values were analyzed by Image J. Except for the anti‐GLIS1 and anti‐PKM2 antibodies purchased from Invitrogen, all other antibodies were purchased from Abcam. All antibodies were diluted according to the manufacturer's instructions.

### 2.16 Immunofluorescence staining

Cells were fixed in 4% paraformaldehyde for 15 min at room temperature, permeabilized with 0.1% Triton X‐100 for 10 min, and blocked with 5% nonfat milk for 1 h. Samples were then incubated overnight at 4°C with a rabbit anti‐GLIS1 primary antibody (Abcam, ab12345, 1:200), followed by Alexa Fluor 488‐conjugated secondary antibody (Abcam, ab150113, 1:500) for 1 h at room temperature. Nuclei were counterstained with 4′,6‐Diamidino‐2‐Phenylindole. Imaging was conducted using a Carl Zeiss AG LSM880 confocal microscope.

### 2.17 Data analysis

All data were obtained from at least three independent experiments and are presented as the mean ± Standard Deviation. Student's *t* tests were applied to examine the differences between two groups. Differences among multiple groups were analyzed using one‐way Analysis of Variance, followed by Tukey's post hoc test. Statistical analyses were performed using the GraphPad Prism 7 software. *p* values <0.05 indicate statistical significance.

## 3 RESULTS

### 3.1 TRIM27 was significantly downregulated in the kidney tissues of RIRI‐induced AKI mice and H_2_O_2_‐treated mRTECs

**FIGURE 1:**
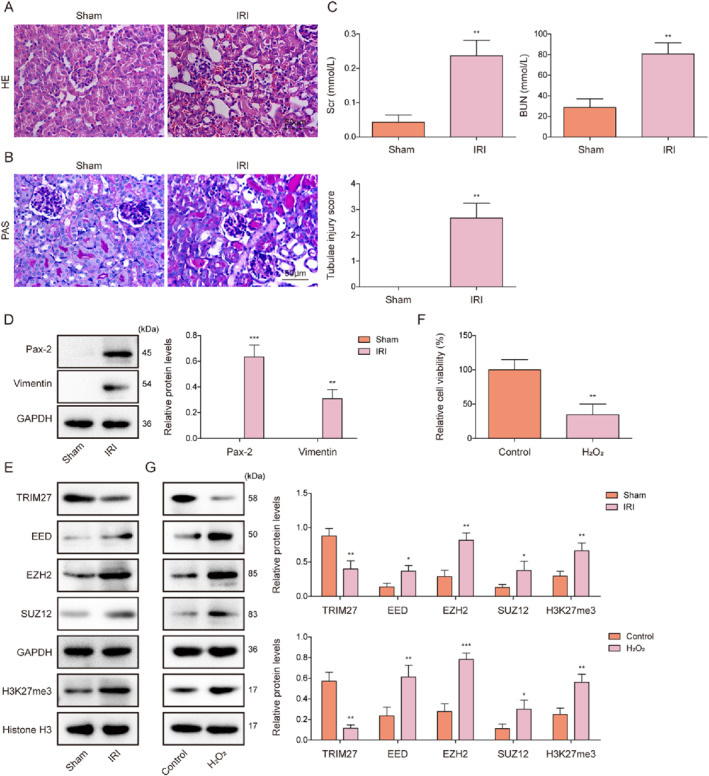
TRIM27 was significantly downregulated in the kidney tissues of I/R‐induced AKI mice and H_2_O_2_‐treated mRTECs. An animal model of RIRI‐induced AKI was established using IRI. (A–B) Pathological changes in the kidney were analyzed by HE and PAS staining, and the injury score is presented. (C) Serum creatinine and BUN levels were examined using the corresponding kits. (D–E) Protein levels of Pax‐2, Vimentin, EED, EZH2, SUZ12, H3K27me3, and TRIM27 in the kidney tissues were determined by western blotting. A renal tubular epithelial cell injury model was established by H_2_O_2_ treatment. (F) The CCK‐8 assay was used to assess mRTEC viability. (G) TRIM27, EED, EZH2, SUZ12, and H3K27me3 protein levels in the cells were examined by western blotting. *N* = 6. *p < 0.05, **p < 0.01, and ***p < 0.001. AKI, acute kidney injury; BUN, blood urea nitrogen; CCK‐8, cell counting kit‐8; EED, Embryonic Ectoderm Development; EZH2, Enhancer of zeste homolog 2; H3K27me3, Histone H3 trimethylated at lysine 27; HE, hematoxylin–eosin; mRTECs, mouse renal tubular epithelial cells; PAS, periodic acid–Schiff; RIRI, renal ischemia‐reperfusion (I/R) injury; SUZ12, Suppressor of Zeste 12; TRIM27, tri‐domain proteins 27.

An animal model of RIRI‐induced AKI was established using IRI. The results of HE and PAS staining showed that IRI induced various pathological changes in the kidney, including inflammatory cell infiltration, tubular structural changes, and an increased renal tubular injury score (Figure 1A and 1B). It was subsequently observed that I/R significantly increased serum Scr and BUN levels in mice (Figure 1C), indicating the successful establishment of an animal model of RIRI‐induced AKI. Dedifferentiation of RTECs is the first step in the renal regeneration response.^24^ As shown in Figure 1D, the dedifferentiation markers (Pax‐2 and Vimentin) were expressed in the kidney tissues of RIRI mice. Further, the levels of EED, EZH2, SUZ12, and H3K27me3 were significantly increased in the kidney tissues of RIRI mice, whereas TRIM27 protein levels were reduced (Figure 1E). An RTEC injury model was subsequently established by H_2_O_2_ treatment. H_2_O_2_ stimulation markedly decreased the viability of mRTECs (Figure 1F). Additionally, H_2_O_2_ treatment significantly reduced TRIM27 protein levels and increased EED, EZH2, SUZ12, and H3K27me3 protein levels in mRTECs (Figure 1G). Collectively, TRIM27 expression was significantly reduced during RIRI‐induced AKI progression.

### 3.2 TRIM27 overexpression enhanced the dedifferentiation, proliferation, and migration of mRTECs while inhibiting apoptosis after H_2_O_2_ treatment

**FIGURE 2:**
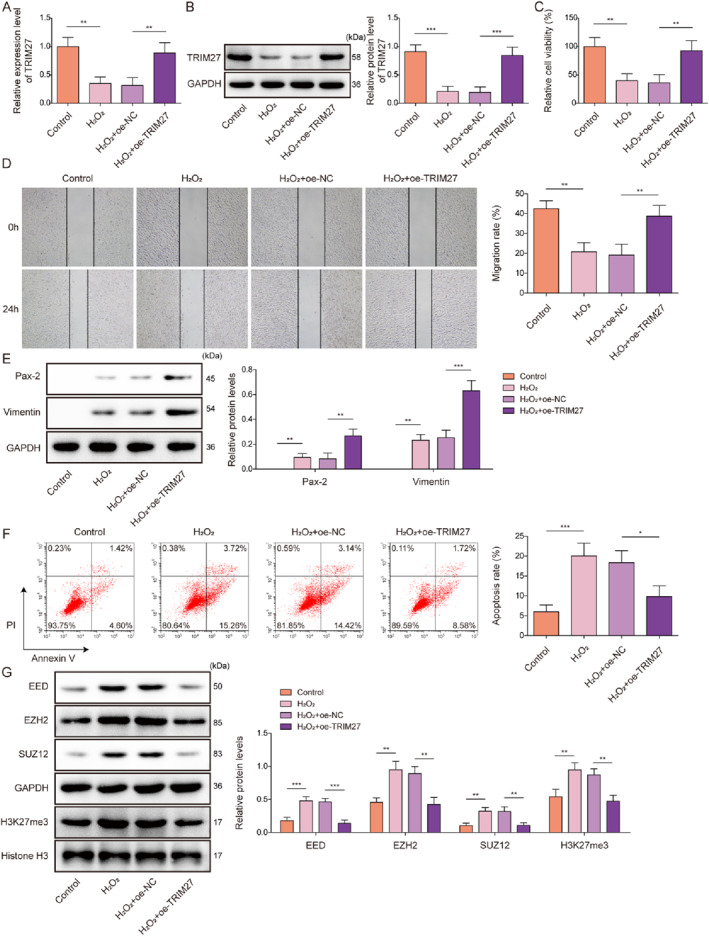
TRIM27 overexpression enhanced the dedifferentiation, proliferation, and migration of mRTECs, while inhibiting apoptosis following H_2_O_2_ treatment. mRTECs were treated with H_2_O_2_ and transfected with Oe‐NC or Oe‐TRIM27. (A–B) TRIM27 expression levels in mRTECs were assessed by qRT‐PCR and western blotting. (C) The CCK‐8 assay was applied to determine viability of mRTECs. (D) Migration of mRTECs was examined using a wound healing assay. (E) Western blotting was performed to analyze Pax‐2 and Vimentin protein levels in mRTECs. (F) Apoptosis was examined using flow cytometry. (G) EED, EZH2, SUZ12, H3K27me3, and Histone H3 in the cells were detected by western blotting. All data were obtained from at least three replicates. *p < 0.05, **p < 0.01, and ***p < 0.001. CCK‐8, cell counting kit‐8; EED, Embryonic Ectoderm Development; EZH2, Enhancer of zeste homolog 2; H3K27me3, Histone H3 trimethylated at lysine 27; mRTECs, mouse renal tubular epithelial cells; qRT‐PCR, quantitative real‐time polymerase chain reaction; SUZ12, Suppressor of Zeste 12; TRIM27, tri‐domain proteins 27.

To investigate the role of TRIM27 in the regulation of mRTEC dedifferentiation after AKI, mRTECs were treated with H_2_O_2_ and subsequently transfected with either Oe‐NC or Oe‐TRIM27. The transfection efficiency of Oe‐TRIM27 was shown in Figure S1A and B, and the results showed that Oe‐TRIM27 transfection significantly increased the mRNA and protein levels of TRIM27 in mRTECs, indicating the successful overexpression of TRIM27. We subsequently observed that H_2_O_2_ treatment significantly impaired TRIM27 expression in mRTECs, which was partially eliminated by Oe‐TRIM27 transfection (Figure 2A and 2B). mRTEC viability and migration were markedly impaired by H_2_O_2_ stimulation, whereas the effects of H_2_O_2_ were weakened by TRIM27 upregulation (Figure 2C and 2D). Notably, TRIM27 overexpression increased the protein levels of dedifferentiation markers Pax‐2 and Vimentin in H_2_O_2_‐treated mRTECs, further enhancing the effects of H_2_O_2_ treatment (Figure 2E). Moreover, TRIM27 overexpression ameliorated the H_2_O_2_‐induced increase in mRTEC apoptosis (Figure 2F). TRIM27 overexpression further reduced EED, EZH2, SUZ12, and H3K27me3 protein levels in H_2_O_2_‐treated mRTECs, which weakened the effects mediated by H_2_O_2_ (Figure 2G). In examining the phenotypic alterations induced by H_2_O_2_, we next evaluated KIM‐1, a well‐characterized biomarker of tubular injury that is known to be upregulated in dedifferentiated, proliferating tubular epithelial cells during kidney repair.^25^ Consistent with this established role, H_2_O_2_ treatment significantly upregulated KIM‐1 expression in cultured RTECs, while this effect was substantially attenuated by TRIM27 overexpression (Figure S1C). Previous studies have demonstrated that hypoxia‐induced glycolytic activation serves as a compensatory energy supply mechanism in acute kidney injury (AKI).^26^ However, prolonged glycolysis may lead to lactate accumulation, inflammatory responses, and fibrosis,^27^ highlighting the critical importance of precise glycolytic regulation in disease progression and recovery. To investigate whether TRIM27 influences mRTEC function via metabolic reprogramming, we examined key glycolytic and oxidative phosphorylation genes upon TRIM27 overexpression. Our results showed that H_2_O_2_ treatment significantly increased glycolytic enzymes (HK2, PKM2, LDHA, and GLUT1) while inhibiting OXPHOS‐related genes (NDUFS1, COX4I1, and ATP5A1), but these changes were eliminated by TRIM27 overexpression (Figure S1D). These results indicate that TRIM27 overexpression enhances mRTECs dedifferentiation, proliferation, and migration while inhibiting apoptosis and excessive glycolysis after AKI.

### 3.3 TRIM27 inhibited PRC2 activity by mediating EZH2 ubiquitination degradation

**FIGURE 3:**
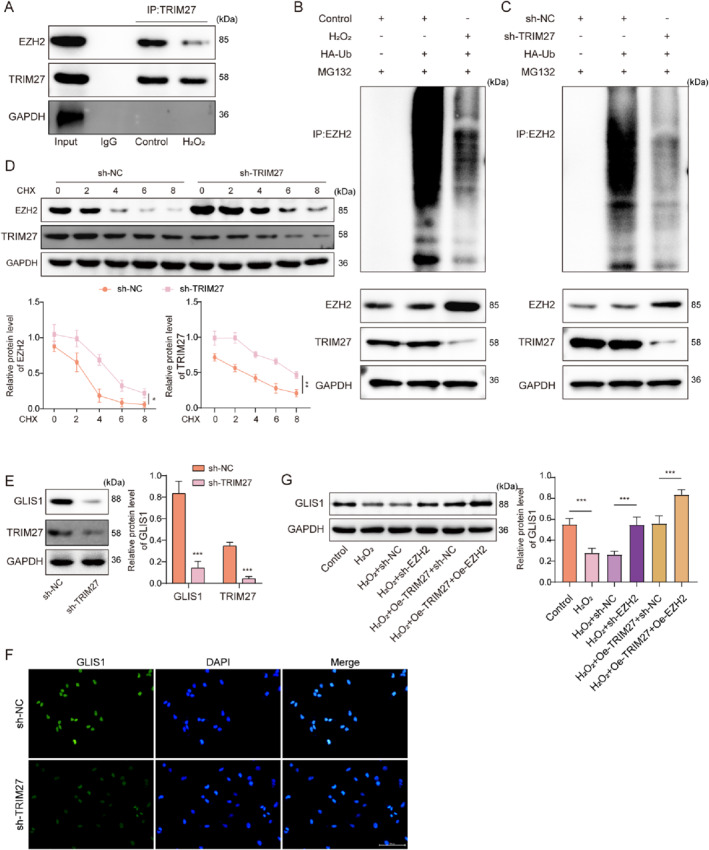
TRIM27 inhibited PRC2 activity by mediating EZH2 ubiquitination degradation. (A) Co‐IP was applied to analyze the interaction between TRIM27 and EZH2 following H_2_O_2_ treatment. (B) The ubiquitination level of the EZH2 promoter in mRTECs after H_2_O_2_ treatment was analyzed by Co‐IP assay. mRTECs were transfected with sh‐NC or sh‐TRIM27. (C) Co‐IP assay was applied to analyze the ubiquitination level of EZH2 in mRTECs. (D) EZH2 and TRIM27 protein degradation in mRTECs following CHX treatment were assessed by western blot. (E) Western blot was adopted to determine GLIS1 and TRIM27 protein levels in mRTECs. (F) The location and expression of GLIS1 in mRTECs were determined by immunofluorescence staining. (G) Both TRIM27 overexpression and EZH2 knockdown were induced in H_2_O_2_‐treated mRTECs, and GLIS1 protein level in cells was detected by western blot. All data were obtained from at least three replicate experiments. *p < 0.05, **p < 0.01, and ***p < 0.001. CHX, cycloheximide; Co‐IP, coimmunoprecipitation; EZH2, Enhancer of zeste homolog 2; mRTECs, mouse renal tubular epithelial cells; PRC2, polycomb repressive complex 2; TRIM27, tri‐domain proteins 27.

TRIM27 can mediate the ubiquitination and degradation of target proteins.^28^ EZH2 is a core subunit of PRC2, which has been identified a key risk factor of RIRI.^9^ Coimmunoprecipitation results showed that TRIM27 interacted with EZH2, but that this interaction was weakened following H_2_O_2_ treatment (Figure 3A). Moreover, H_2_O_2_ treatment reduced TRIM27 protein levels and EZH2 ubiquitination, while increasing EZH2 protein levels in mRTECs (Figure 3B). TRIM27 knockdown reduced the ubiquitination level of EZH2 and increased its protein expression in mRTECs (Figure 3C). TRIM27 silencing reduced the degradation of EZH2 and TRIM27 proteins in cycloheximide‐treated mRTECs (Figure 3D). It also turned out that sh‐TRIM27 transfection significantly decreased GLIS1 and TRIM27 protein levels in mRTECs (Figure 3E). Meanwhile, as revealed by immunofluorescence staining, GLIS1 was mainly located in the nucleus, and TRIM27 knockdown significantly reduced GLIS1 expression in mRTECs (Figure 3F). As revealed in Figure 3G, H_2_O_2_ treatment significantly reduced GLIS1 protein expression in mRTECs, while either EZH2 silencing or TRIM27 overexpression alone upregulated GLIS1 protein expression. Moreover, the combination of TRIM27 overexpression and EZH2 silencing further elevated GLIS1 protein level (Figure 3G). Taken together, these results indicated that TRIM27 can mediate the ubiquitination degradation of EZH2.

### 3.4 TRIM27 regulated H_2_O_2_‐treated mRTEC dedifferentiation, proliferation, migration, and apoptosis by targeting PRC2

**FIGURE 4:**
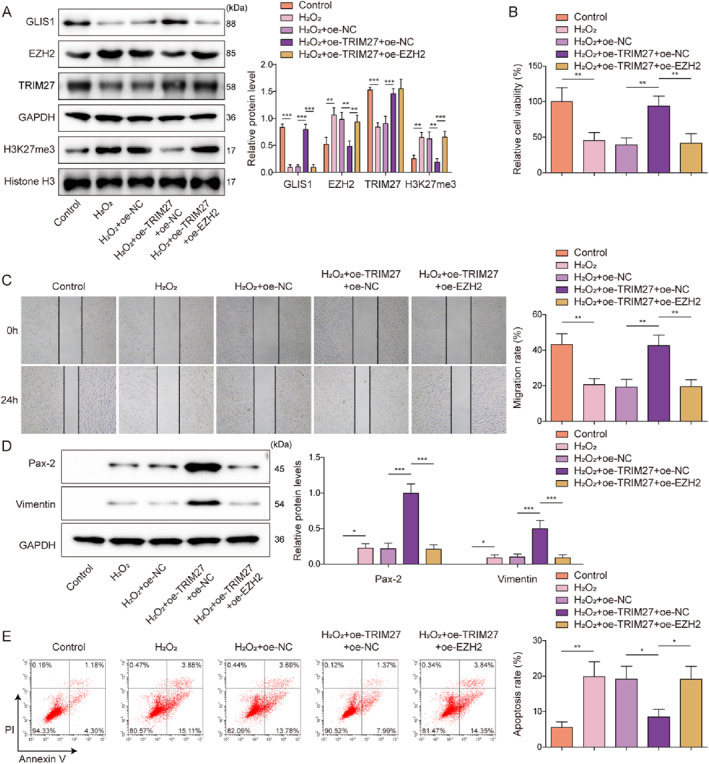
TRIM27 regulated H_2_O_2_‐treated mRTEC dedifferentiation, proliferation, migration, and apoptosis by targeting PRC2. Overexpression of both TRIM27 and EZH2 was induced in H_2_O_2_‐treated mRTECs. (A) TRIM27, EZH2, GLIS1, H3K27me3, and Histone H3 protein levels in mRTECs were assessed using western blotting. (B) Viability of mRTECs was examined using the CCK‐8 assay. (C) A wound healing assay was conducted to analyze mRTEC migration. (D) Western blotting was performed to detect Pax‐2 and Vimentin protein levels in mRTECs. (E) Flow cytometry was used to detect apoptosis. All the data were obtained from at least three replicates. *p < 0.05, **p < 0.01, and ***p < 0.001. CCK‐8, cell counting kit‐8; EZH2, Enhancer of zeste homolog 2; H3K27me3, Histone H3 trimethylated at lysine 27; mRTECs, mouse renal tubular epithelial cells; PRC2, polycomb repressive complex 2; TRIM27, tri‐domain proteins 27.

To investigate the interaction between TRIM27 and EZH2 in the regulation of mRTEC dedifferentiation after AKI, overexpression of both TRIM27 and EZH2 was induced in H_2_O_2_‐treated mRTECs. As shown in Figure 4A, Oe‐TRIM27 transfection ameliorated the H_2_O_2_‐induced increase in EZH2 and H3K27me3 protein levels in mRTECs, which was attenuated following co‐transfection with Oe‐TRIM27 and Oe‐EZH2. Meanwhile, TRIM27 overexpression upregulated GLIS1 protein expression in H_2_O_2_‐treated mRTECs, whereas co‐overexpression of TRIM27 and EZH2 led to a decrease in GLIS1 protein level (Figure 4A). It was also observed that Oe‐TRIM27 transfection prevented H_2_O_2_‐induced decrease in TRIM27 protein level in mRTECs, and Oe‐EZH2 transfection had no significant effect on TRIM27 level in cells (Figure 4A). Subsequently, the promoting effect of TRIM27 overexpression on H_2_O_2_‐treated mRTECs viability, migration, and the protein levels of dedifferentiation markers (Pax‐2 and Vimentin), as well as the inhibitory effect on apoptosis, were weakened by EZH2 overexpression (Figure 4B–E). In summary, TRIM27 overexpression enhanced mRTECs dedifferentiation, proliferation, and migration, while inhibiting apoptosis following AKI through the regulation of EZH2 expression.

### 3.5 PRC2 inactivated the Wnt/β‐catenin pathway by promoting GLIS1 DNA methylation

**FIGURE 5:**
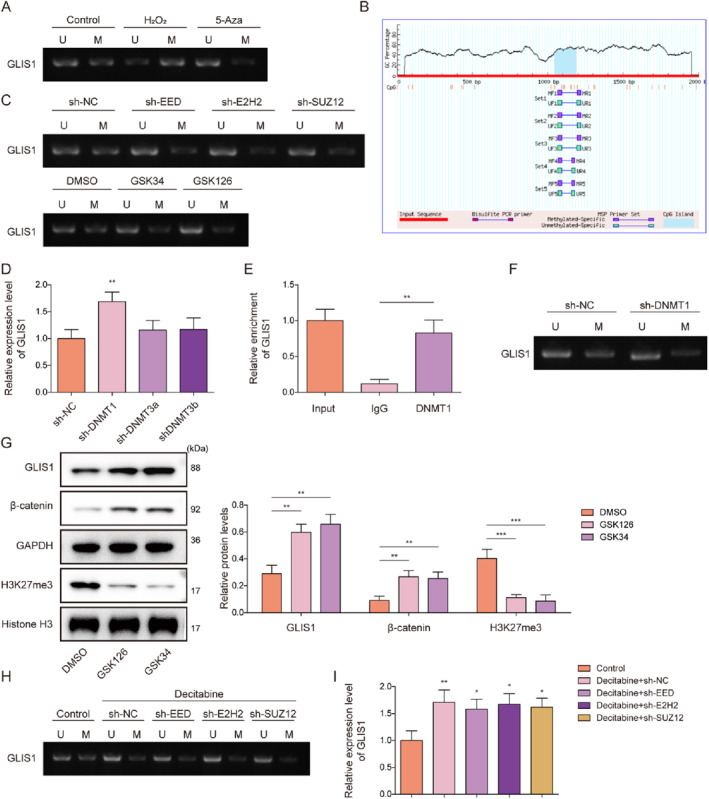
PRC2 inactivated the Wnt/β‐catenin pathway by promoting GLIS1 DNA methylation. (A) The methylation level of the GLIS1 promoter in mRTECs after H_2_O_2_ treatment was analyzed using an MSP assay. (B) The existence of CpG islands in the GLIS1 promoter was predicted using MethPrimer. (C) We knocked down the subunits of PRC2 (EZH2, EED, and SUZ12) in mRTECs or treated cells with PRC2 inhibitors (GSK126 and GSK343), after which the methylation level of the GLIS1 promoter in mRTECs was analyzed by MSP assay. (D) GLIS1 mRNA level in mRTECs after sh‐NC, sh‐DNMT1, sh‐DNMT3A, or sh‐DNMT3B transfection was examined using qRT‐PCR. (E) ChIP assay was employed to analyze the interaction between DNMT1 and GLIS1. (F) The methylation level of the GLIS1 promoter in mRTECs was examined after sh‐NC or sh‐DNMT1 transfection using MSP assay. (G) Western blot was employed to monitor the protein levels of GLIS1, H3K27me3, Histone H3, and β‐catenin in mRTECs after treatment with PRC2 inhibitors (GSK126 and GSK343); treatments were assessed using western blot. We knocked down the subunits of PRC2 (EZH2, EED, and SUZ12) in mRTECs combined with Decitabine treatment. (H) MSP assay was performed to detect the methylation level of the GLIS1 promoter in mRTECs. (I) qRT‐PCR was employed to examine GLIS1 mRNA levels in mRTECs. All data were obtained from at least three replicate experiments. *p < 0.05, **p < 0.01, and ***p < 0.001. ChIP, chromatin immunoprecipitation; EED, Embryonic Ectoderm Development; EZH2, Enhancer of zeste homolog 2; H3K27me3, Histone H3 trimethylated at lysine 27; mRTECs, mouse renal tubular epithelial cells; MSP, methylation‐specific PCR; PRC2, polycomb repressive complex 2; qRT‐PCR, quantitative real‐time polymerase chain reaction; SUZ12, Suppressor of Zeste 12.

PRC2 catalyzes H3K27 methylation of the target gene promoter through the methyltransferase EZH2, whereas EED serves as an epigenetic “reader”, and SUZ12 serves as the scaffold for the complex.^8^ There results were identified the key downstream effector of PRC2 in RIRI. Multiple genes, including GLIS1, TRAF1, and MG53, have been reported to exert protective effects in kidney injury.^2023^, ^2025^, ^2023^, ^2015^ We performed chromatin immunoprecipitation (ChIP) assays and found that DNMT1 prominently enriched at the GLIS1 and TRAF1 promoters (Figure S1E, Figure 5E). However, TRAF1 promoter methylation was unaffected by EZH2 knockdown (Figure S1F). Therefore, GLIS1 was selected for mechanistic investigation. It was also observed that the methylation level of the GLIS1 promoter in mRTECs was markedly increased by H_2_O_2_ treatment and abrogated by treatment with the DNA methyltransferase (DNMT) inhibitor 5‐Aza (Figure 5A). MethPrimer predicted the presence of CpG islands in the GLIS1 promoter (Figure 5B). To investigate the effect of PRC2 on GLIS1 DNA methylation, we knocked down the subunits of PRC2 (EZH2, EED, and SUZ12) in mRTECs, or treated the cells with PRC2 inhibitors (GSK126 and GSK343). The results showed that the GLIS1 promoter showed low methylation in mRTECs following EZH2, EED, and SUZ12 knockdown, or PRC2 inhibitor treatment (Figure 5C), indicating that PRC2 activity was required for GLIS1 promoter hypermethylation. DNA methylation is regulated by several DNMTs, including DNMT1, DNMT3A, and DNMT3B.^32^ We observed that DNMT1 knockdown significantly increased GLIS1 mRNA levels in mRTECs, whereas knockdown of DNMT3A and DNMT3B had no significant effects on GLIS1 mRNA levels (Figure 5D). A ChIP assay revealed that DNMT1 interacted with the GLIS1 promoter (Figure 5E). As expected, DNMT1 knockdown markedly reduced methylation of the GLIS1 promoter in mRTECs (Figure 5F). PRC2 is the only known methyltransferase with H3K27 activity (H3K27me1, H3K27me2, and H3K27me3), while H3K27me3 has been shown to be involved in mediating the DNA methylation of gene promoters.^12^ Our results indicated that treatment with the PRC2 inhibitors (GSK126 and GSK343) increased GLIS1 protein levels and reduced H3K27me3 levels in mRTECs (Figure 5G). GLIS1 is a known upstream activator of the Wnt/β‐catenin pathway.^33^ We observed that treatment with the PRC2 inhibitors GSK126 and GSK343 increased the β‐catenin protein level in mRTECs (Figure 5G). To further investigate the relationship between DNA methylation and PRC2‐mediated regulation of GLIS1 expression by H3K27me3, mRTECs were further subjected to Decitabine (a broad‐spectrum DNMT inhibitor) to study the link between DNA methylation and PRC2‐mediated regulation of GLIS1 expression by H3K27me3. As presented in Figure 5H and 5I, Decitabine treatment significantly reduced the methylation level of the GLIS1 promoter and the increased GLIS1 mRNA levels in mRTECs. Above results remained consistent even when the PRC2 subunits were knocked down separately. In conclusion, GLIS1 expression in mRTECs was regulated by H3K27me3 and DNA methylation. Additionally, PRC2‐mediated H3K27me3 activation was required for GLIS1 promoter DNA methylation.

The key downstream effector of PRC2 in RIRI was identified. Multiple genes, including GLIS1, TRAF1, and MG53, have been reported to exert protective effects in kidney injury.^2023^, ^2025^, ^2023^, ^2015^ We performed chromatin immunoprecipitation (ChIP) assays and found that DNMT1 prominently enriched at the GLIS1 and TRAF1 promoters (Figure S1E). However, TRAF1 promoter methylation was unaffected by EZH2 knockdown (Figure S1F). Therefore, GLIS1 was selected for mechanistic investigation. It was also observed that the methylation level of the GLIS1 promoter in mRTECs was markedly increased by H_2_O_2_ treatment and abrogated by treatment with the DNA methyltransferase (DNMT) inhibitor 5‐Aza (Figure 5A). MethPrimer predicted the presence of CpG islands in the GLIS1 promoter (Figure 5B). To investigate the effect of PRC2 on GLIS1 DNA methylation, we knocked down the subunits of PRC2 (EZH2, EED, and SUZ12) in mRTECs, or treated the cells with PRC2 inhibitors (GSK126 and GSK343). The results showed that the GLIS1 promoter showed low methylation in mRTECs following EZH2, EED, and SUZ12 knockdown, or PRC2 inhibitor treatment (Figure 5C), indicating that PRC2 activity was required for GLIS1 promoter hypermethylation. DNA methylation is regulated by several DNMTs, including DNMT1, DNMT3A, and DNMT3B.^32^ We observed that DNMT1 knockdown significantly increased GLIS1 mRNA levels in mRTECs, whereas knockdown of DNMT3A and DNMT3B had no significant effects on GLIS1 mRNA levels (Figure 5D). A ChIP assay revealed that DNMT1 interacted with the GLIS1 promoter (Figure 5E). As expected, DNMT1 knockdown markedly reduced methylation of the GLIS1 promoter in mRTECs (Figure 5F). PRC2 is the only known methyltransferase with H3K27 activity (H3K27me1, H3K27me2, and H3K27me3), while H3K27me3 has been shown to be involved in mediating the DNA methylation of gene promoters.^12^ Our results indicated that treatment with the PRC2 inhibitors (GSK126 and GSK343) increased GLIS1 protein levels and reduced H3K27me3 levels in mRTECs (Figure 5G). GLIS1 is a known upstream activator of the Wnt/β‐catenin pathway.^33^ We observed that treatment with the PRC2 inhibitors GSK126 and GSK343 increased the β‐catenin protein level in mRTECs (Figure 5G). To further investigate the relationship between DNA methylation and PRC2‐mediated regulation of GLIS1 expression by H3K27me3, mRTECs were further subjected to Decitabine (a broad‐spectrum DNMT inhibitor) to study the link between DNA methylation and PRC2‐mediated regulation of GLIS1 expression by H3K27me3. As presented in Figure 5H and 5I, Decitabine treatment significantly reduced the methylation level of the GLIS1 promoter and the increased GLIS1 mRNA levels in mRTECs. These results were consistent remained knocking down the PRC2 subunits separately. In conclusion, GLIS1 expression in mRTECs was regulated by H3K27me3 and DNA methylation. Additionally, PRC2‐mediated H3K27me3 activation was required for GLIS1 promoter DNA methylation.

### 3.6 PRC2 regulated the dedifferentiation, proliferation, migration, and apoptosis of mRTECs after H_2_O_2_ treatment by GLIS1

**FIGURE 6:**
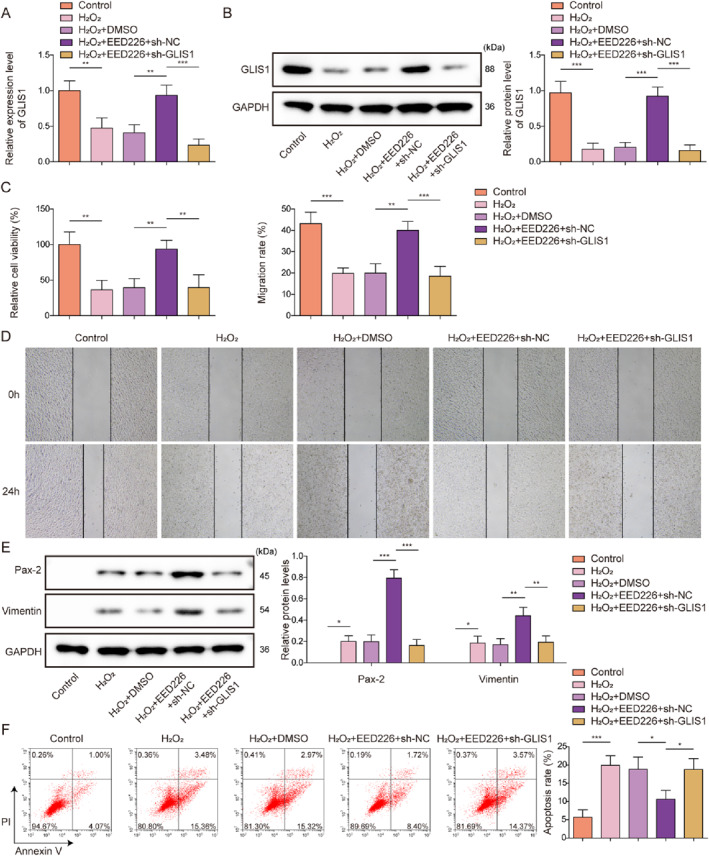
Inhibition of PRC2 activity enhanced the dedifferentiation, proliferation, and migration of mRTECs, while inhibiting apoptosis followed H_2_O_2_ treatment through the regulation of GLIS1. H_2_O_2_‐treated mRTECs were treated with a PRC2 inhibitor (EED226), and transfected with sh‐NC or sh‐GLIS1. (A, and B) The mRNA and protein levels of GLIS1 in mRTECs were assessed using qRT‐PCR and western blotting, respectively. (C) Viability of mRTECs was analyzed using the CCK‐8 assay. (D) A wound healing assay was performed to detect mRTECs migration. (E) Western blotting was applied to detect Pax‐2 and Vimentin protein levels in mRTECs. (F) Apoptosis was assessed by flow cytometry. All data were obtained from at least three replicates. *p < 0.05, **p < 0.01, and ***p < 0.001. CCK‐8, cell counting kit‐8; EED226, Embryonic Ectoderm Development 226; mRTECs, mouse renal tubular epithelial cells; PRC2, polycomb repressive complex 2; qRT‐PCR, quantitative real‐time polymerase chain reaction.

To investigate the roles of PRC2 and GLIS1 in regulating mRTEC dedifferentiation after AKI, H_2_O_2_‐treated mRTECs were treated with a PRC2 inhibitor (Embryonic Ectoderm Development 226, EED226) and subsequently transfected with sh‐NC or sh‐GLIS1. As shown in Figure 6A and 6B, H_2_O_2_ treatment significantly reduced GLIS1 expression level in mRTECs, which was abrogated by EED226 treatment, an effect, which was weakened by sh‐GLIS1 transfection. EED226 treatment markedly promoted H_2_O_2_‐treated mRTEC viability and migration, as well as the protein levels of the dedifferentiation markers Pax‐2 and Vimentin in cells, while inhibiting apoptosis (Figure 6C–F). We further observed that these effects of EED226 were partially reversed by GLIS1 knockdown (Figure 6C–F). Collectively, GLIS1 knockdown weakened the effects of PRC2 inhibition on mRTEC dedifferentiation, proliferation, migration, and apoptosis following AKI.

### 3.7 Knockdown of GLIS1 reversed the promoting effect of TRIM27 overexpression on the dedifferentiation, proliferation, and migration of mRTECs

To investigate the roles of GLIS1 and TRIM27 in regulating mRTEC dedifferentiation after AKI, H_2_O_2_‐treated mRTECs were co‐transfected with Oe‐TRIM27 and sh‐GLIS1. It was firstly observed that the inhibitory effect of Oe‐TRIM27 transfection on H3K27me3 levels in mRTECs and the promoting effect on GLIS1 were attenuated following co‐transfection with Oe‐TRIM27 and sh‐GLIS1 (Figure S2A). It was also observed that Oe‐TRIM27 transfection prevented H_2_O_2_‐induced decrease in TRIM27 protein level in mRTECs, and sh‐GLIS1 transfection had no significant effect on TRIM27 level in cells (Figure S2A). It was subsequently observed the promoting effect of TRIM27 overexpression on H_2_O_2_‐treated mRTECs viability, migration, and the protein levels of dedifferentiation markers (Pax‐2 and Vimentin) were weakened by GLIS1 silencing (Figure S2B–D). In summary, TRIM27 overexpression enhanced the dedifferentiation, proliferation, and migration of mRTECs by upregulating GLIS1.

### 3.8 TRIM27 overexpression ameliorated I/R‐induced AKI in mice

**FIGURE 7:**
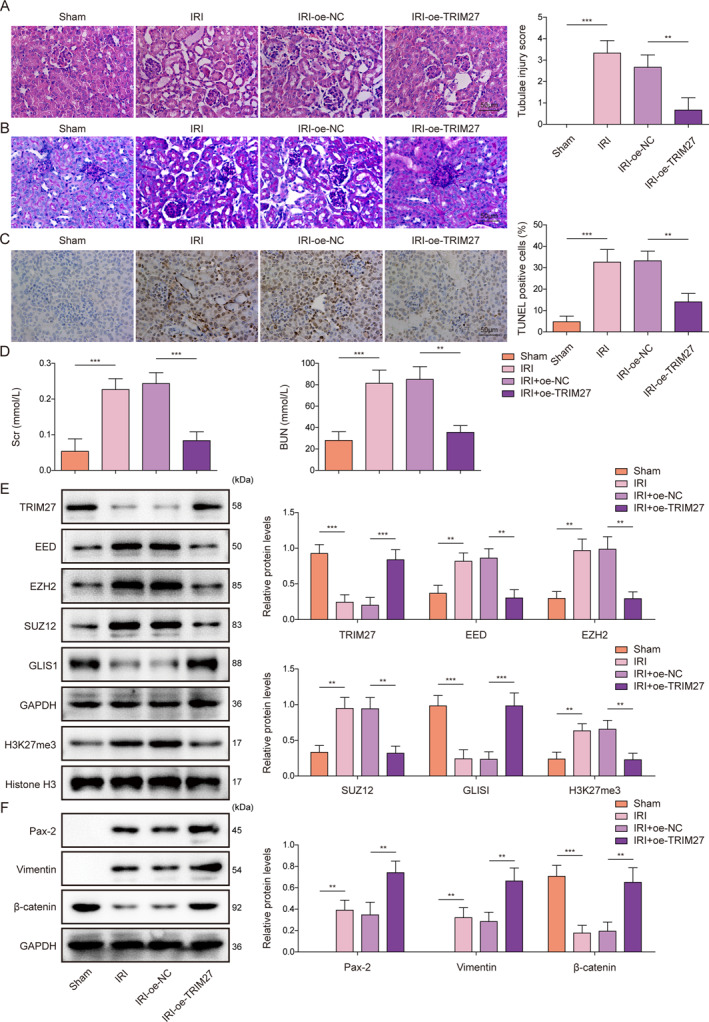
TRIM27 overexpression ameliorated I/R‐induced AKI in mice. An animal model was established using I/R combined with Oe‐TRIM27 injection. (A, B) Hematoxylin and eosin (HE) and periodic acid–Schiff (PAS) staining were performed to analyze the pathological changes in the kidneys. (C) Apoptosis in the kidney tissues was analyzed by TUNEL staining. (D) Serum creatinine and BUN levels were measured using the corresponding kits. (E–F) Western blot was employed to detect TRIM27, EED, EZH2, SUZ12, GLIS1, H3K27me3, Histone H3, Pax‐2, Vimentin, and β‐catenin protein levels in kidney tissues. *N* = 6. *p < 0.05, **p < 0.01, and ***p < 0.001. AKI, acute kidney injury; BUN, blood urea nitrogen; EED, Embryonic Ectoderm Development; EZH2, Enhancer of zeste homolog 2; H3K27me3, Histone H3 trimethylated at lysine 27; HE, hematoxylin and eosin; I/R, ischemia‐reperfusion; PAS, periodic acid–Schiff; SUZ12, Suppressor of Zeste 12; TRIM27, tri‐domain proteins 27; TUNEL, terminal deoxynucleotidyl transferase‐mediated dUTP nick‐end labeling.

To further investigate the role of TRIM27 in the regulation of RIRI‐induced AKI in vivo, an RIRI mouse model was established. HE and PAS staining demonstrated that renal pathological changes in RIRI mice were ameliorated by TRIM27 overexpression (Figure 7A and 7B). Apoptosis in kidney tissues was markedly facilitated after IRI and alleviated by TRIM27 overexpression (Figure 7C). In addition, Oe‐TRIM27 injection markedly reduced serum Scr and BUN levels in RIRI mice (Figure 7D). Further, TRIM27 overexpression significantly reduced TRIM27 and GLIS1 protein levels while elevating EED, EZH2, SUZ12, and H3K27me3 levels in the kidney tissues of RIRI mice (Figure 7E). We further observed that TRIM27 overexpression increased the levels of Pax‐2, Vimentin, and β‐catenin in the kidney tissues of RIRI mice (Figure 7F). In summary, TRIM27 overexpression ameliorates RIRI‐induced AKI in mice by enhancing mRTEC dedifferentiation.

## 4 DISCUSSION

RIRI, a complex and poorly understood process, is the leading cause of AKI and is associated with rapid renal insufficiency and high mortality.^34^ Despite progress in the development of supportive treatments and prevention methods, the mortality rate among patients remains high.^35^ Therefore, there is an urgent need to develop novel therapeutic strategies for RIRI‐induced AKI. Our primary findings in the present study were that TRIM27 upregulation promoted mRTEC dedifferentiation, proliferation, and migration by promoting GLIS1 expression and inhibiting PRC2 activity, thus alleviating RIRI‐induced AKI.

Dedifferentiation of RTECs is the first step in the renal regeneration response.^24^ In mature adult kidneys, RTECs differentiate efficiently, and do not express markers such as Pax‐2 and Vimentin, which are only expressed in mesenchymal cells.^36^ Nevertheless, many RTECs expressing these molecules have been found in proximal renal tubules after IRI.^24^ Consistently, our results showed that protein levels of several dedifferentiation markers (Pax‐2 and Vimentin) were increased in the kidney tissues of RIRI mice and H_2_O_2_‐treated mRTECs, accompanied by impaired proliferation and migration abilities. However, the molecular mechanisms underlying RTEC dedifferentiation after injury remain unknown. TRIM27 is a member of the TRIM family, consisting of three conserved domains and a highly variable C‐terminal region.^37^ TRIM27 is closely associated with cell proliferation, apoptosis, and inflammation.^2019^, ^2020^ Notably, prior research has demonstrated that TRIM27 overexpression alleviates AKI in mice by reducing inflammation and apoptosis in the kidney tissues.^19^ However, the role of TRIM27 in the regulation of RTEC dedifferentiation during RIRI‐induced AKI development remains unclear. In the present study, TRIM27 overexpression improved mRTEC injury following RIRI‐induced AKI by enhancing mRTECs dedifferentiation. These results indicate that TRIM27 upregulation mitigates RIRI‐induced AKI by promoting the dedifferentiation, proliferation, and migration of mRTECs, while inhibiting apoptosis. Glycolysis, as the fundamental metabolic pathway for glucose breakdown, plays complex roles in cellular responses to injury and disease. In ischemic diseases, hypoxia‐induced glycolysis initially acts as an adaptive response, but excessive lactate accumulation can result in acidotoxicity and cell death.^26^ Notably, a previous study showed that TRIM27 promotes glycolysis to drive malignant proliferation in cancer cells.^40^ Our results showed that TRIM27 overexpression significantly ameliorated H_2_O_2_‐induced increase in glycolytic enzymes (HK2, PKM2, LDHA, and GLUT1) and prevented H_2_O_2_‐induced decrease in OXPHOS‐related genes (COX4I1, ATP5A1, and NDUFS1). These results suggest TRIM27 promotes RTEC proliferation and RIRI recovery by inhibiting excessive glycolysis, revealing that the regulatory relationship between TRIM27 and glycolysis varies under AKI condition.

It has been widely reported that PRC2 plays an important role in AKI development, and may be a therapeutic target for AKI.^11^ From a molecular function perspective, PRC2 establishes inhibitory states for certain genes during AKI development.^41^ EZH2, a subunit of the PRC2 complex, plays an important role in AKI development.^2018^, ^2022^ EZH2 inhibition alleviates RIRI‐induced AKI by inhibiting apoptosis, inflammation, and fibrosis in the kidney tissue.^2023^, ^2019^ Our findings also demonstrate that EZH2 is highly expressed in the kidney tissues of IRI mice and H_2_O_2_‐treated mRTECs. TRIM27 plays a role in diseases by inducing the ubiquitination of downstream targets.^28^ Our findings show, for the first time, that TRIM27 inhibits PRC2 activity by mediating EZH2 ubiquitination and degradation. As expected, EZH2 upregulation inhibited the effects of TRIM27 overexpression on H_2_O_2_‐treated mRTECs dedifferentiation, proliferation, and migration. Collectively, our results show that TRIM27 upregulation mitigated RIRI‐induced AKI by promoting mRTEC dedifferentiation, proliferation, and migration, while inhibiting apoptosis by mediating EZH2 ubiquitination and degradation.

As previously reported,^12^ PRC2 mediates SFRP1 promoter DNA methylation and H3K27me3 to suppress its expression, and PRC2 exacerbates RIRI injury. Therefore, we hypothesized that downstream genes regulated by PRC2 might play protective roles in RIRI. Through literature screening, we found that GLIS1, TRAF1, and MG53 exerts protective effects in the kidney.^2023^, ^2025^, ^2023^, ^2015^ ChIP data confirmed DNMT1's significant enrichment at GLIS1 and TRAF1 loci. However, EZH2 knockdown did not notably affect TRAF1 methylation, prompting us to prioritize GLIS1 as the key target. GLIS1 is a Kruppel‐like zinc‐finger transcription factor that induces stem cell pluripotency.^44^ Additionally, GLIS1 is involved in the regulation of cystic renal disease progression.^45^ Notably, GLIS1 alleviates renal fibrosis during kidney aging.^15^ In the present study, we observed that H_2_O_2_ treatment increased the methylation level of the GLIS1 promoter, and reduced its expression in mRTECs. PRC2 may regulate DNA methylation by affecting the activity of DNMTs, which may include PRC2 subunits.^12^ In the present study, our results showed that the knockdown of PRC2 subunits (EZH2, SUZ12, and EED) or treatment with PRC2 inhibitors (GSK126 and GSK343) reduced the methylation level of the GLIS1 promoter in mRTECs. As previously reported, epigenetic control of both H3K27me3 and DNA methylation influence target gene expression.^46^ PRC2 is the only known methyltransferase, which also exerts H3K27 activity, while its catalytic subunit, EZH2, requires EED and SUZ12 for its catalytic activity.^47^ As reported, EZH2, as the catalytic subunit of PRC2, can mediate H3K27me3 to repress transcription^48^ and also interact with DNMTs to facilitate CpG island methylation.^49^ Our findings show that GLIS1 expression in mRTECs is regulated by both H3K27me3 and DNA methylation. Additionally, PRC2‐mediated H3K27me3 activation was required for GLIS1 promoter DNA methylation. As expected, GLIS1 knockdown reversed the effect of the PRC2 inhibitor (EED226) on H_2_O_2_‐treated mRTECs dedifferentiation, proliferation, and migration. Collectively, PRC2 impairs mRTEC dedifferentiation, proliferation, and migration by regulating GLIS1 DNA methylation and H3K27me3 levels. The Wnt/β‐catenin signaling is a complex and evolutionary conserved pathway that plays a role in kidney damage and healing following various stressors.^50^ The activation of the Wnt/β‐catenin signaling pathway alleviates AKI.^51^ The previous studies demonstrated that the Wnt/β‐catenin pathway facilitated RTEC dedifferentiation under high glucose conditions.^2020^, ^2015^ In addition, it was previously reported that Wnt/β‐catenin pathway activation improved renal function and reduced tubular injury after ischemia/reperfusion by preventing RTEC apoptosis and promoted cell proliferation.^54^ These findings underscore the close association between the Wnt/β‐catenin pathway and RTEC functionality. Notably, GLIS1 has been identified as an upstream activator of Wnt/β‐catenin signaling.^33^ Herein, we observed that PRC2 inactivated the Wnt/β‐catenin signaling by mediating GLIS1 DNA methylation and H3K27me3. Moreover, it was observed that knockdown of TRIM27 suppressed GLIS1 expression in mRTECs, and overexpression of TRIM27 upregulated both GLIS1 and β‐catenin expression. Importantly, our experimental results indicated that TRIM27 and GLIS1 collectively promoted RTEC dedifferentiation, proliferation, and migration. These observations lead us to preliminarily hypothesize that activation of the Wnt/β‐catenin pathway may play a positive role in RTEC dedifferentiation or other cellular functions. However, the specific underlying mechanisms require further investigation. Referring to previous reports;^2023^, ^2024^ we selected 24 h of reperfusion as the optimal experimental condition to establish the IRI mouse model. Meanwhile, our results indicated that there was obvious tissue injury in the kidney tissue of mice after IRI for 24 h, and the serum levels of Scr and BUN were significantly increased. This result further proved that 24 h was a suitable time period for constructing the IRI model. Importantly, a previous study^56^ showed that the serum levels of Scr and BUN in rats reached a peaked 24 h after IRI, and began to decline 48 h later. Therefore, we did not conduct further experiments with IRI at 48 and 72 h. However, in the future, if conditions permit, we will further investigate the changes in the expression of TRIM27, GLIS1, and PRC2 at different IRI treatment times. In addition, our western blot analysis revealed a consistent 2.3‐fold upregulation of H3K27me3 levels in both RIRI mice and H_2_O_2_‐treated mRTECs, with quantification normalized to the histone H3 reference, representing relative rather than absolute measurements. However, direct comparison of H3K27me3 levels between in vivo and in vitro systems is biologically problematic due to inherent disparities^1^: Whole‐kidney lysates encompass non‐epithelial cell types (e.g., interstitial and immune cells) that likely dilute tubular‐specific signals, whereas cultured mRTECs represent a homogeneous population amplifying detectable changes^2^; The in vivo microenvironment (e.g., oxygen gradients and paracrine signaling) and the coexistence of epithelial cells at distinct states (proliferating, senescent, or apoptotic) introduce layer‐specific regulatory dynamics absent in monolayer cultures. Importantly, our bulk western blot approach cannot resolve this cellular heterogeneity—particularly the divergent fates of injured tubular cells, where functional repair competes with maladaptive senescence/apoptosis. While single‐cell RNA sequencing (scRNA‐Seq) could delineate H3K27me3's role in these subpopulations, technical constraints currently limit its application in our study, warranting future investigation when feasible.

Taken together, our results showed that TRIM27 reduced GLIS1 DNA methylation and increased its expression, subsequently activating the Wnt/β‐catenin pathway by inhibiting PRC2 activity through mediating EZH2 ubiquitination degradation, thereby promoting mRTECs dedifferentiation, proliferation, and migration and ultimately alleviating RIRI‐induced AKI (graphical abstract). Our results indicate that TRIM27 plays a crucial role in renal regeneration following RIRI‐induced AKI and may be a potential therapeutic target for RIRI‐induced AKI.

## AUTHOR CONTRIBUTIONS

Chongxiang Xiong and Shougang Zhuang designed this study. Chongxiang Xiong, Haishan Chen, Baoting Su, Li Zhang, Jingxiang Hu, and Qiaowen Wang collected the materials and performed the experiments. Chongxiang Xiong analyzed the data and wrote the manuscript. Shougang Zhuang revised the manuscript. All authors read and approved the final version of the manuscript.

## CONFLICT OF INTEREST STATEMENT

The authors declare no conflicts of interest.

## ETHICS STATEMENT

The animal studies were approved by The First Dongguan Affiliated Hospital of Guangdong Medical University.

## Supporting information

Supporting Information S1

Figure S1

Figure S2

## Data Availability

All data generated or analyzed during this study are included in this published article.
