# Supplementary material for: Tri‐domain proteins 27 alleviates ischemia‐reperfusion injury‐induced acute kidney injury by promoting Gli‐like transcription factor 1 expression via the inhibition of polycomb repressive complex 2 activity
Source: J Cell Commun Signal. 2025 Sep 18;19(3):e70046. doi: 10.1002/ccs3.70046 (PMC12444411; doi:10.1002/ccs3.70046)
Supplement: Supplementary file 1 — Supporting Information S1 [file CCS3-19-e70046-s001.docx]

**Supplemental figure legends**

**Supplemental Figure 1.** (A-B) mRTECs were transfected with Oe-NC or Oe-TRIM27, and the mRNA and protein levels of TRIM27 in mRTECs were assessed by qRT-PCR and western blotting. mRTECs were treated with H_2_O_2_ and transfected with Oe-NC or Oe-TRIM27. (C) KIM-1 protein level in cells was examined by western blotting. (D) The protein levels of TRIM27, HK2, PKM2, LDHA, GLUT1, COX4I1, ATP5A1, and NDUFS1 in cells were examined by western blotting. (E) ChIP assay was employed to detect the bindings relationship between TRAF1/MG53 and DNMT1. (F) The methylation level of the TRAF1 promoter in mRTECs after sh-NC or sh-EZH2 transfection was analyzed using an MSP assay. All data were obtained from at least three replicates. **p* < 0.05, ***p* < 0.01, ****p* < 0.001.

**Supplemental Figure 2. Knockdown of GLIS1 reversed the promoting effect of TRIM27 overexpression on the dedifferentiation, proliferation, and migration of mRTECs.**

H_2_O_2_-treated mRTECs were co-transfected with Oe-TRIM27 and sh-GLIS1. (A) TRIM27, GLIS1, H3K27me3, and Histone H3 protein levels in mRTECs were assessed using western blotting. (B) Viability of mRTECs was examined using the CCK-8 assay. (C) A wound healing assay was conducted to analyze mRTEC migration. (D) Western blotting was performed to detect Pax-2 and Vimentin protein levels in mRTECs. All the data were obtained from at least three replicates. **p* < 0.05, ***p* < 0.01, ****p* < 0.001.
