# Supplementary figures and images for: Tri‐domain proteins 27 alleviates ischemia‐reperfusion injury‐induced acute kidney injury by promoting Gli‐like transcription factor 1 expression via the inhibition of polycomb repressive complex 2 activity
Source: J Cell Commun Signal. 2025 Sep 18;19(3):e70046. doi: 10.1002/ccs3.70046 (PMC12444411; doi:10.1002/ccs3.70046)

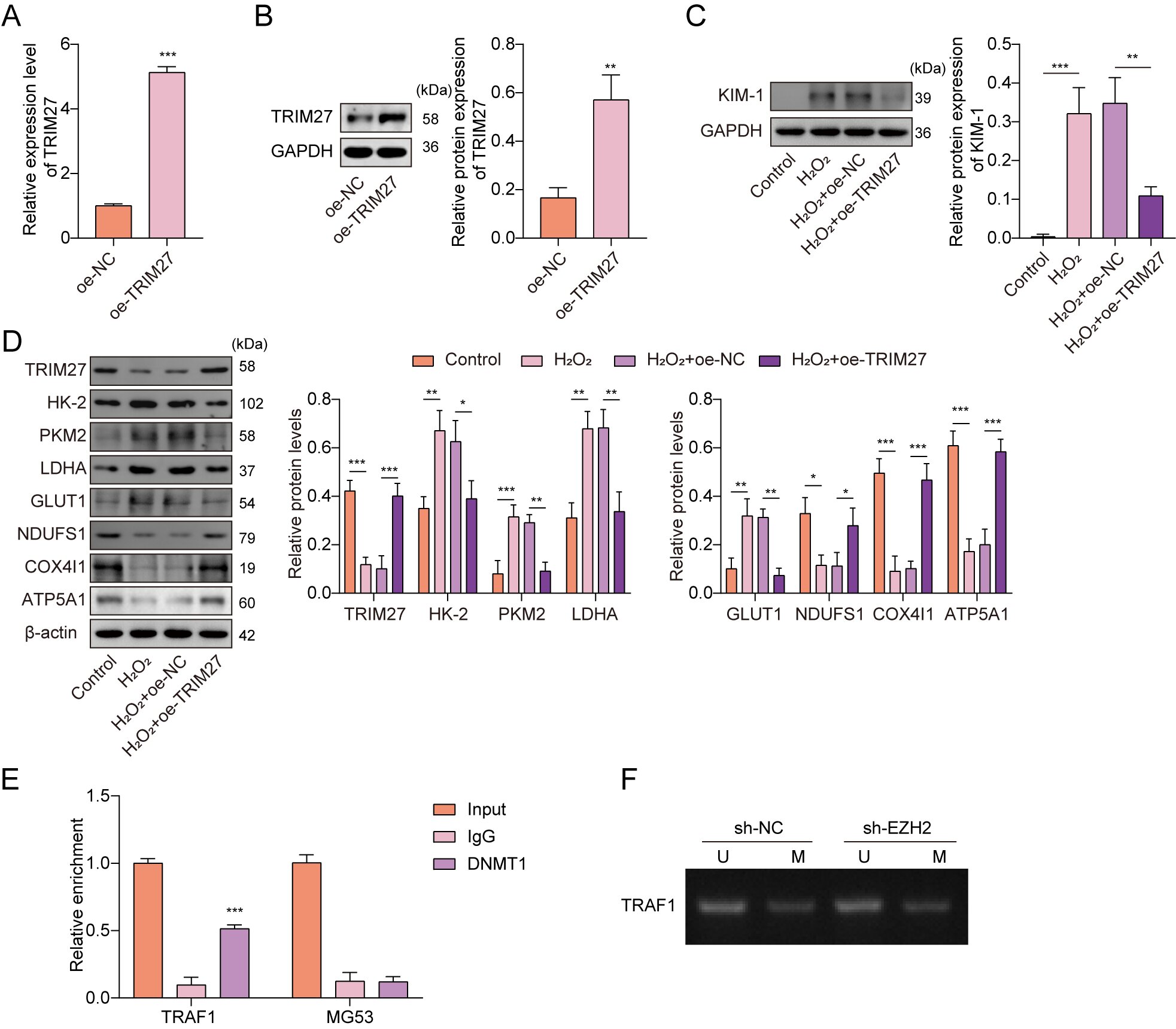

Supplement: Supplementary file 2 — Figure S1 [file CCS3-19-e70046-s003.jpg]

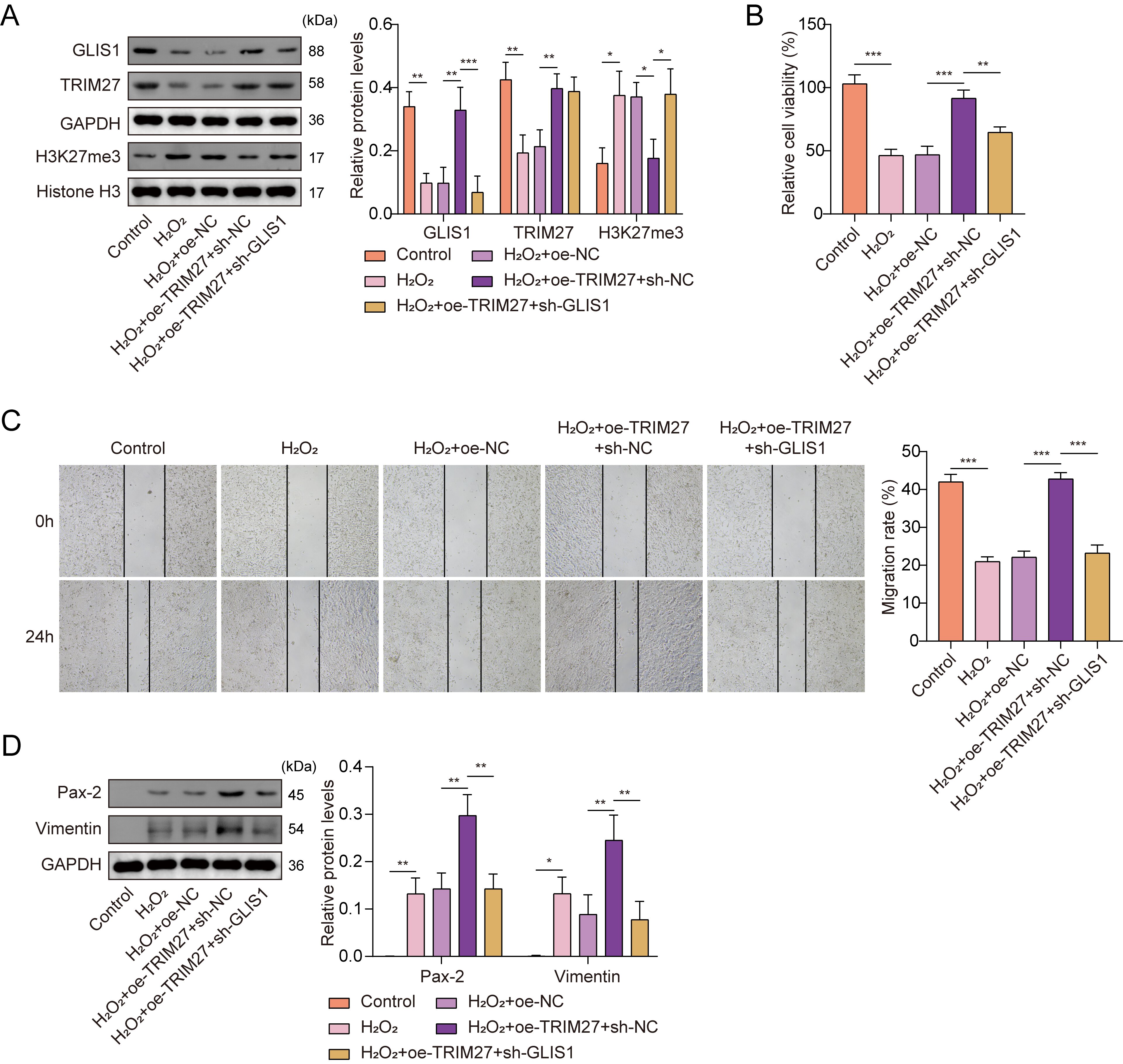

Supplement: Supplementary file 3 — Figure S2 [file CCS3-19-e70046-s002.jpg]
